# Sleep disturbances among Chinese clinical nurses in general hospitals and its influencing factors

**DOI:** 10.1186/s12888-017-1402-3

**Published:** 2017-07-03

**Authors:** Hongyun Dong, Qiong Zhang, Zihua Sun, Fengxin Sang, Yingzhi Xu

**Affiliations:** Shouguang People’s Hospital, NO. 45, Jiankang Street, Shouguang, Weifang, Shandong Province China

**Keywords:** Sleep disturbance, Nurse, Stress, Risk factor, Epidemiology

## Abstract

**Background:**

This study aimed to determine the prevalence of sleep disturbances among clinical nurses in general hospitals in Mainland China, and identify its associate factors.

**Methods:**

Using a cross-sectional design, a total of 5012 clinical nurses selected by random cluster sampling completed the survey on the Pittsburgh Sleep Quality Index (PSQI), measures of quality of life indexed by the Medical Outcomes Study 12-item Short-Form Health Survey, occupational stress evaluated by the Job Content Questionnaire, lifestyle and sociodemographic details.

**Results:**

The average PSQI score of 4951 subjects was 7.32 ± 3.24, including 3163 subjects with PSQI ≥5, accounting for 63.9%. Multivariate Logistic regression analysis showed that the risk factors for sleep disturbances in nurses were female gender, the Emergency department and ICU, many years of service, high night shift frequency, professional status: primary and intermediate, employment status: temporary, poor quality of life: poor mental health, low perceived health, high occupational stress (high psychological demand, low job control and low workplace social support).

**Conclusions:**

Sleep disturbances are highly prevalent among clinical nurses in general hospitals in Mainland China. Many of the factors listed above were associated with the prevalence of sleep disturbances in nurses, and occupational stress plays an important role in the development of sleep disturbances in Chinese clinical nurses.

## Background

As it is known, sleep is a necessary part of our life. Sleep difficulties are ubiquitous and a common complaint among the general population in Western countries, with rates of self-reported insomnia ranging between 10 and 48% [1–4]. Long-term sleep problems could lead to serious effects such as thought retardation, memory loss, slow response, low spirit, irritability and even the increase in the possibility of depression and suicidal tendency [5, 6]. Chronic sleep problems have also been associated with greater work absenteeism [7] and work-related accidents or injuries [4, 8–10].

Nurses, as a special occupational group, bear relatively high mental stress and a large amount of job tasks in caring for patients [11, 12]. Moreover, clinical nurses need to keep an all-night vigil. This working mode makes their sleep time irregular [13–15]. The sleep quality problem of nurses has become a prominent social focus [16]. Studies have shown that sleep disturbances in nurses not only influences their own health, but also affects nursing quality and even the psychological health and treatment process of patients [17]. However, there has been limited research about current sleep disturbance status among clinical nurses in Mainland China. At present, little is known about the risk factors of their sleep disturbances.

Occupational stress, one of the environmental factors, is believed to be a significant factor in many diseases [18–23]. Clinical nurses have stressful jobs, are frequently on rotating work shifts, undergo emotional stress, and work long hours every day due to their job requirements [24–27]. Therefore, a certain correlation between stress and sleep disturbances among clinical nurses was hypothesized.

Our study analyzed the present sleep disturbance status among clinical nurses working in general hospitals in Mainland China, the differences in the sleep disturbances of nurses with different gender, age, marital status, educational background, professional status, employment status, years of service and departments, and factors that influence sleep disturbances in nurses. The relevance between sleep disturbances and occupational stress in nurses was also analyzed, in order to provide a basis for improving the sleep quality of nurses, relieve their occupational stress, and promote their physical and psychological health.

## Methods

### Sample

Considering data collection quality and availability, six general hospitals were selected by random cluster sampling from among all tertiary referral hospitals (38 tertiary referral hospitals in total) in Shandong Province, China. Among the nurses in the above six hospitals, whole clinical nurses who have worked for at least 1 year were selected and interviewed. Exclusion criteria were as follows: nurses (679 in total) whose sleep was affected by other causes such as drinking tea (at least once a day), drinking liquor (at least once a day), disease (heart failure, hyperthyroidism, heartburn, restless leg syndrome, chronic pain, etc.), family history of sleep disorders and taking medicines. Considering the convenience and economic efficiency during the survey, 367 nurses who took a marriage leave, maternity leave, sick leave, and leave for personal affairs were not included. A total of 5012 clinical nurses completed our questionnaire from May 2015 to December 2015. The study was approved by the Ethics Committee of Shouguang People’s Hospital. All participants in the study were voluntary and provided a written informed consent before participating into this survey. However, 61 questionnaires that lacked the key variables or were filled in irregularly were eliminated. Therefore, 4951 subjects were included.

### Survey questionnaire

The self-administered questionnaire comprised of four sections, and it took the subjeccts approximately 20 min to complete.

### Sleep disturbances

The Pittsburgh Sleep Quality Index (PSQI) [28] was employed in the present study to estimate the prevalence of sleep disturbances. The PSQI evaluates multiple dimensions of sleep over a 1-month period [29–31]. Nineteen individual items generate seven component scores: subjective sleep quality, sleep duration, sleep latency, habitual sleep efficiency, sleep disturbances, the use of sleeping medications, and daytime dysfunction [29]. The seven component scores were summed for one global score of subjective sleep quality (range: 0–21), and higher scores represent poorer subjective sleep quality [29]. The Chinese version of the PSQI has good overall reliability (*r* = 0.82–0.83) and test–retest reliability (*r* = 0.77–0.85) [28]. A PSQI global score > 5 yielded a sensitivity of 98% and a specificity of 55%, as a marker for poor sleep in primary sleep disturbance sufferers vs. healthy controls [28]. Respondents were classified as having sleep disturbances if they obtained a PSQI global score > 5 [28–31].

### Health-related quality of life (QoL) and measuring scales

The Chinese version of the 12-item Short Form Health Survey (SF-12) was used to measure QoL. SF-12 is a practical scale for large group comparisons with a focus on overall physical and mental health outcomes [32–34]. Researches have indicated that the Chinese version of SF-12 is an effective measurement tool for the Chinese population [35]. It is feasible and credible [36]. The present study used two components from the scale in the current analysis: Mental Component Summary (MCS) score and Physical Component Summary (PCS) score. These scores were based on weighting responses to all 12 items, with higher scores indicating better QoL.

### Sociodemographic characteristics and occupational factors

Sociodemographic and occupational data were gathered on sex, age, education level, marital status, employment status (permanent vs. temporary), departments, number of night shifts per month, years of service, and professional status (registered, primary, intermediate, and senior/deputy senior).

### Occupational stress

Occupational stress was assessed by a validated Chinese version questionnaire [37–39]: Job Content Questionnaire (JCQ). The 22-item JCQ, based on the Job Demand Control Support model [40], consists of three dimensions: psychological demand (five items), job control including skill discretion (six items) and decision-making authority (three items), and workplace social support including coworker social support (four items) and supervisor social support (four items) [38, 41]. For each item, the response was recorded using a four-point Likert scale ranging from 1 (strongly disagree) to 4 (strongly agree). The Chinese version of JCQ is a reliable and valid tool for measuring job stressors of the Chinese occupational groups [38]. In the study, Cronbach’s α coefficient for the psychological demand subscale was 0.75, while those for the job control and workplace social support subscales were 0.87 and 0.86, respectively.

### Statistical analysis

Statistical analysis was performed using SPSS version 18.0. Differences between with and without sleep disturbances were examined by single-factor chi-square test (for categorical variables) and independent t-test (for continuous variables). Odds ratios (ORs) and 95% confidence intervals (95% CI) were calculated to examine the association of sleep disturbances with occupational, psychological, QoL and demographic factors using multivariate logistic regression analysis. Initially, univariate analyses were calculated, with each of the potential explanatory variables as independent variables, and sleep disturbances as the dependent variable. Pre-selection for the entry of all associated factors into the multivariate logistic regression model required a *P*-value of <0.05 in the univariate analyses. A 5% significance level was accepted for all tests.

## Results

### General conditions

The average age of the 4951 nurses was 28 ± 6 years old, including 284 male subjects (5.74%) and 4667 female subjects (94.26%). The average PSQI score was 7.32 ± 3.24, including 3163 subjects with a PSQI ≥5, accounting for 63.9%.

### Comparison of different research subjects with and without sleep disturbances

Table 1 shows that sleep disturbances were statistically related to age, gender, department, years of service, night shift frequency per month, professional status and employment status (*P* < 0.05). Both marital status and educational background were not statistically related to sleep disturbances in nurses.

**Table 1:** Comparison of different research subjects with sleep disturbances

Factor	NO. of subjects	Subjects with PSQI > 5	Prevalence (%)	χ2	*p*
Gender	4951	3163	63.9	8.15	0.004
Male	284	159	56.0		
Female	4667	3004	64.4		
Age				183.836	<0.001
20–29	1932	1057	54.7		
30–39	1455	934	64.2		
40–49	987	689	69.8		
50 and above	577	483	83.7		
Marital status				1.534	0.215
Married	3187	2016	63.3		
Unmarried	1764	1147	65.0		
Years of service				159.765	<0.001
1–9	1893	1012	53.5		
10–19	1421	948	66.7		
20–29	953	705	74.0		
≥30	684	498	72.8		
Educational background				2.629	0.453
Lower than junior college	1269	792	62.4		
Junior college	2063	1314	63.7		
Bachelor	987	643	65.1		
Master or above	632	414	65.5		
Night shift frequency (per month)				29.279	<0.001
Few	831	468	56.3		
General	1921	1224	63.7		
Many	2199	1471	66.9		
Department				14.751	0.022
Internal medicine	1752	1101	62.8		
Surgical department	1307	811	62.1		
of pediatrics	431	278	64.5		
of gynecology and obstetrics	373	237	63.5		
Emergency department	605	412	68.1		
ICU	264	189	71.6		
Operating room	219	135	61.6		
Employment status				14.028	<0.001
Permanent	2483	1523	61.3		
Temporary	2468	1640	66.5		
Professional status				10.37	0.016
Registered nurse	1069	671	62.8		
Primary	2127	1408	66.2		
Intermediate	934	589	63.1		
Senior/deputy senior	821	495	60.3		

### Quality of life factors associated with sleep disturbances

Results of the univariate analyses revealed that the mean scores of SF12-PCS, SF12-MCS, and perceived health in the past 3 months between subjects with and without sleep disturbances were statistically different (*P* < 0.05, Table 2).

**Table 2:** Relationship between occupational stress, quality of life and sleep disturbances among clinical nurses

Factor	Sufferers		Non-sufferers			
	Mean	Standard deviation	Mean	Standard deviation	*t*	*P*
JCQ						
Skill discretion	17.23	2.16	15.54	2.78	23.775	<0.001
Decision-making authority	9.75	2.49	11.16	2.95	−17.880	<0.001
Psychological job demands	16.26	3.78	12.23	4.75	32.770	<0.001
Supervisor social support	11.27	2.73	14.19	3.05	−34.632	<0.001
Coworker social support	11.91	2.29	13.24	2.97	−17.583	<0.001
Quality of life						
SF12-PCS	41.57	6.87	42.29	8.03	−3.329	<0.001
SF12-MCS	35.34	6.21	37.51	6.38	−11.694	<0.001
Perceived health in the past 3 months	1.62	0.64	2.11	0.66	−25.585	<0.001

### Relevance between sleep disturbances and occupational stress

In terms of the JCQ, the mean scores of decision-making authority, skill discretion, psychological demand, supervisor social support and coworker social support between subjects with and without sleep disturbances were statistically different (*P* < 0.05, Table 2).

### Logistic regression analysis of multiple factors influencing sleep disturbances

Table 3 shows that sleep disturbances revealed an independent relevance with gender, department, years of service, professional status, night shift frequency per month, employment status, QoL (mental health), perceived health in the past 3 months, decision-making authority, skill discretion, psychological demand, supervisor social support and coworker social support. In other words, the risk factors for sleep disturbances in nurses were female gender, ICU and Emergency Department, many years of service, high night shift frequency, professional status: primary and intermediate, employment status: temporary, poor QoL: poor mental health, low perceived health, high occupational stress (high psychological demand, low job control and low workplace social support).

**Table 3:** Logistic regression analysis of multiple factors influencing sleep disturbances

Variables and assignment	B	S.E.	*P*	OR	95% C.I. for OR	
					Lower	Upper
Gender (1 = male, 2 = female)	0.478	0.197	<0.001	1.613	1.096	2.373
Years of service: 1-9				1.000		
10-19	0.486	0.118	0.016	1.626	1.290	2.049
20-29	0.613	0.122	0.003	1.846	1.453	2.345
≥ 30	0.499	0.108	0.007	1.647	1.333	2.035
Night shift frequency per month: Few				1.000		
General	0.425	0.131	0.017	1.530	1.183	1.977
Many	0.621	0.115	<0.001	1.861	1.485	2.331
Department: Internal medicine				1.000		
Surgical department	−0.126	0.121	0.575	0.882	0.695	1.118
of pediatrics	0.109	0.218	0.184	1.115	0.727	1.710
of gynecology and obstetrics	0.113	0.193	0.371	1.120	0.767	1.634
Emergency department	0.429	0.118	0.021	1.536	1.219	1.935
Operating room	0.108	0.131	0.260	1.114	0.862	1.440
ICU	0.795	0.201	0.007	2.214	1.493	3.284
Professional status: Registered nurse				1.000		
Primary	0.509	0.127	<0.001	1.664	1.297	2.134
Intermediate	0.428	0.114	0.011	1.534	1.227	1.918
Senior/deputy senior	−0.227	0.112	0.013	0.797	0.640	0.993
Employment status (1 = permanent, 2 = temporary)	0.337	0.115	<0.001	1.401	1.118	1.755
JCQ						
Skill discretion	0.347	0.117	0.008	1.415	1.125	1.779
Decision-making authority	−0.489	0.105	<0.001	0.613	0.499	0.753
Psychological job demand	0.305	0.098	<0.001	1.357	1.120	1.644
Supervisor social support	−0.307	0.123	<0.001	0.736	0.578	0.936
Coworker social support	−0.268	0.114	<0.001	0.765	0.612	0.956
Quality of life						
SF12-MCS	−0.319	0.137	0.005	0.727	0.556	0.951
Perceived health in the past 3 months	−0.284	0.107	0.013	0.753	0.610	0.928

## Discussion

### Analysis of sleep quality among clinical nurses in general hospitals in mainland China

To our knowledge, this is the first large-scale survey concerning sleep disturbances and associate risk factors of clinical nurses in Mainland China. Participants were sampled from six randomly selected tertiary-level hospitals of different cities in Shandong Province, which improves the generalizability of the results for clinical nurses working in tertiary hospitals in China. The overall prevalence of sleep disturbances in clinical nurses in general hospitals in Mainland China was 63.9%, which was higher than the general population [1, 2, 7, 42–45]. Using structured questions, an earlier population-based study (*n* = 9851) reported that the overall prevalence of insomnia among Hong Kong Chinese was at 11.9%, with females being at higher risk for insomnia than males [44]. Other researchers in Tehran [46] found that 53.1% of health care workers had sleep disturbances measured by PSQI, which was lower than that in our finding. A pilot study in Taiwan using the same standard of PSQI [47] also revealed that nurses were most likely to suffer from sleep disturbances with an OR of 5.51 (95% CI: 2.09-14.51), compared to other hospital staff (physician OR = 1). Therefore, clinical nurses are prone to have sleep disturbances than other health care workers.

In China, clinical nurses in general hospitals receive and treat many patients with critical disease, difficult diseases, and major surgery. As such, for nurses in general hospitals, updating their knowledge hierarchy in time and boosting their clinical practice ability are necessary, which may result in potential pressure on nurses. Therefore, the sleep disturbances of clinical nurses may be the focus of more attention.

### Influence of sociodemographic and QoL factors on sleep disturbances in clinical nurses

In line with existing epidemiological data [44, 48, 49], our findings revealed that female nurses suffered from sleep disturbances more easily than male nurses. This finding may be related to the female physiological and psychological features. In China, female nurses need to work and take care of their family at the same time, such that their life pressure is higher than that of male nurses. Sleep disturbances are frequently symptoms of anxiety and depression, which may be reflected in the sleep quality difference caused by gender difference [48, 50]. Thus, their sleep quality may be affected more easily. Therefore, more attention should be paid to the sleep quality of female nurses.

Meanwhile, nurses with primary and intermediate professional status may suffer from sleep disturbances more easily. The Ministry of Health in China classifies the grade of nurses according to their years of service, working ability and capacity for scientific research, as follows: registered, primary, intermediate, deputy senior, and senior. Primary and intermediate nurses still need to master the specialty proficiently and put in considerable effort for in-depth learning. Meanwhile, primary and intermediate nurses are faced with increasing pressure of further job title evaluation.

Our results replicated existing data that sleep disturbances were associated with impaired QoL [51, 52]. However, in our study, only the mental health component was retained in the final model of regression analyses for sleep disturbances. Poor mental health was associated with sleep disturbances in clinical nurses, which was consistent with previous reports [1, 53–55]. These findings suggest that the pathway that mediated between sleep disturbances and QoL was more likely to be psychological, rather than physical [56].

### Effects of job nature and department on sleep disturbances

In our study, more years of service was significantly associated with a greater prevalence of sleep disturbances, indicating that the job nature of clinical nurses affects the prevalence of sleep disturbances among nursing staff. Other researchers have also revealed that more years of service is a risk factor of sleep disturbances [57]. As service years increases, nurses would shoulder greater work pressure and face a more complicated personal relationship, which could all lead to the attack of sleep disturbances; while the sleep quality of nurses with more than 30 years of service would slightly be better. This may be due to the fact that nurses with more than 30 years of service are going to retire, undertake less job tasks, and do not have to take night shifts.

This research indicates that PSQI scores have significant differences in different departments. The departments that resulted in sleep disturbances among nurses most easily are ICU and the Emergency Department. Nurses in the ICU had the highest risk of sleep disturbances, which may be related to the heavy workload, long-term contact with critical and seriously ill patients, evident occupational exposure, high work intensity and high pressure. For example, considering the particularity of the ICU, family members are not allowed to accompany and attend to patients. Thus, clinical nurses of the ICU bear all basic nursing work. The research of other study [58] also revealed that clinical nurses in the ICU show poor sleep quality, fatigue, drowsiness, anxiety and depression. However, the root causes of these findings are unconfirmed. Nurses in the Emergency Department rank top 2 in terms of the occurrence rate of sleep disturbances. Medical disputes often occur in the Emergency Department in China. Meanwhile, emergency nurses need to care for critically and seriously ill patients for a long time. In the process, their mind is highly stressed, their body is fatigued, and their sleep quality may be affected [59].

Nursing personnel with high night shift frequency per month may suffer from sleep disturbances more easily, which was consistent with the finding of other researchers [42, 60, 61]. Clinical nurses frequently take turns in working night shifts, which disturbs their body clock and results in irregular sleep patterns [62, 63]. With the accelerating development of China’s economy, more and more medical services are needed, especially in general hospitals; and nurses in general hospitals are relatively in shortage, such that the monthly night shift frequency of nurses increases. However, whether the additional workload could affect sleep disturbances in nurses would remain unclear as this study did not study the workload in night shifts. Nursing workload in night shifts may be heavy. Most often, nurses during night shifts work independently and lack group support. As such, nurses are more intense in night shifts than in day shifts. Their mind and body are in the stress state. Thus, their cerebral cortex malfunctions and their biological rhythm are disturbed, which may lead to sleep disturbances in nurses.

Our study also revealed that the sleep quality of temporary nurses was more easily affected than that of permanent nurses. Temporary employees experience more job insecurity than permanent ones [64, 65]. Job insecurity has been identified as an important occupational stress factor [66], which contribute negatively to the psychological and physical health and well-being of employees [67–69]. Temporary nurses, who were more likely to be actually dismissed from work, were more likely to be stressed and work harder, in order to keep their job. Hence, this may lead to sleep disturbances.

### A certain correlation between occupational stress and sleep disturbances

Healthcare professionals, especially nurses, are highly stressed [70–72]. Job strain, a key component of work stress, is a measure of the balance between the psychological demand of a job and the amount of control or decision-making power it affords [18]. In our study, high psychological demand, low job control, and low workplace social support were associated with the development of sleep disturbances, which was similar to another study [73]. We found that scores for psychological demand and skill discretion correlated positively with sleep disturbances, while scores for decision-making authority correlated negatively with sleep disturbances. Working in the hospital is highly stressful, because workers need to deal with unpredictable medical conditions, have excessive workloads and working hours, are exposed to high levels of stress, and are frequently emotionally exhausted [25]. Meanwhile, nurses still need to continuously learn new knowledge, in order to improve their ability, meet job requirements and ensure promotion.

In the present study, clinical nurses sensed a lack of social support from supervisors and peers. In another study [74], work relationships have been proven to be a direct source of stress. Seeking support from coworkers or supervisors may actually represent a health-protecting behavior that could help diffuse the impact of stressors in the workplace [47]. Therefore, measures such as conflict resolution and peer support groups may be provided in tertiary hospitals in China, in order to decrease occupational stress, reduce the development of sleep disturbances and promote health.

### Limitations

The limitations of this study include the use of cross-sectional data and self-report measures. Although there are theoretically sound reasons to assume that the factors mentioned above could affect the sleep quality of nurses, no solid conclusion regarding causal relationships can be made from the data derived from this cross-sectional study. Whether the findings are applicable to all clinical nurses in Mainland China, this could not be assessed by only including one hospital nurse level in the study. The long-term sick leave nurses who did not participate in our study may be affected by sleep disturbances. The study included only QoL measurement and no specific depression or anxiety or other mental health (such as psychiatric disorders or medications) measures, which limited analyzing the relationship of mental health with sleep disturbances. Another limitation is that our study ignored the quantitative interaction between psychological, QoL, occupational and personal factors. Further research needs to consider the relative relationships of intermediate factors to establish sleep disturbances models for clinical nurses.

## Conclusions

This study shows that the overall occurrence rate of sleep disturbances among clinical nurses in general hospitals in Mainland China is high. The risk factors for sleep disturbances in nurses were female gender, ICU and Emergency Department, many years of service, high night shift frequency, professional status: primary and intermediate, employment status: temporary, poor QoL: poor mental health, low perceived health, high occupational stress (high psychological demand, low job control and low workplace social support). Occupational stress plays an important role in the development of sleep disturbances in Chinese clinical nurses. In addition, sleep disturbances among clinical nurses have been a major psychological health problem in China, which needs to be solved urgently.

## Abbreviations

JCQ: Job Content Questionnaire
MCS: The Mental Component Summary
OR: Odds ratio
PCS: The Physical Component Summary
PSQI: Pittsburgh Sleep Quality Index
QoL: Quality of life
SF-12: The 12-item Short Form Health Survey
